# Role of PBN‐CeA Circuit in Paclitaxel‐Induced Neuropathic Pain and Negative Emotions

**DOI:** 10.1002/cns.71065

**Published:** 2026-08-27

**Authors:** Huiqin Chen, Liang Chen, Yu Chen, Yin Huang, Fei Fan, YiQian Liu, Qianli Zhang, Ying Tang, Aiqin Chen, Chun Lin

**Affiliations:** ^1^ Pain Research Institute, School of Basic Medical Sciences, Fujian Provincial Key Laboratory of Brain Aging and Neurodegenerative Diseases Fujian Medical University Fuzhou Fujian People's Republic of China; ^2^ School of Basic Medical Sciences Key Laboratory of Translational Tumor Medicine in Fujian Province, Putian University Putian China; ^3^ Fujian Health College Fuzhou Fujian People's Republic of China

**Keywords:** amygdala, dynorphin–κ opioid receptor, negative emotion, neuropathic pain, parabrachial nucleus

## Abstract

**Background:**

Paclitaxel produces a chronic, glove‐stocking neuropathy that is frequently accompanied by anxiety and depression. However, the neural circuits and molecular signals driving this pain–affect comorbidity remain unclear.

**Methods:**

Chemotherapy‐induced neuropathic pain (CINP) was established by paclitaxel injections in male rats. Mechanical and thermal sensitivity and negative emotional behaviors were assessed using behavioral tests. Parabrachial nucleus (PBN)–central amygdala (CeA) projections were traced with viral vectors, and optogenetics was used to manipulate PBN terminals. Prodynorphin (PDYN) and κ‐opioid receptor (KOR) mRNA levels were quantified by reverse transcription quantitative polymerase chain reaction (RT‐qPCR).

**Results:**

CINP rats developed persistent pain hypersensitivity and negative emotional behaviors, accompanied by increased CeA c‐Fos and PDYN expression without changes in KOR mRNA. Intra‐CeA administration of the KOR antagonist Navacaprant alleviated both pain and negative emotional behaviors. Optogenetic inhibition of the PBN–CeA pathway reduced CeA activation, attenuated pain hypersensitivity, and improved negative emotional behaviors, whereas pathway activation elicited both changes in naïve rats. Chronic inhibition of this pathway lowered CeA PDYN mRNA levels in CINP rats.

**Conclusion:**

The PBN‐CeA pathway contributes to paclitaxel‐induced neuropathic pain and anxiety‐like behavior, and is associated with increased dynorphin expression in the CeA. Targeting this pathway or its downstream KOR may provide a therapeutic strategy for chemotherapy‐related sensory and affective disturbances.

## Graphical abstract

Paclitaxel‐induced neuropathy recruits the PBN–CeA pathway and increases CeA PDYN expression, contributing to pain hypersensitivity and negative emotional behaviors. Suppressing PBN inputs to the CeA or blocking KOR with Navacaprant mitigates both sensory hypersensitivity and negative emotional symptoms.

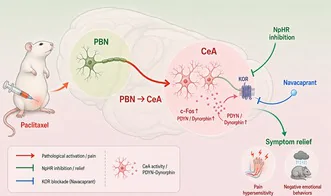

## 1 Introduction

Paclitaxel is a frontline chemotherapeutic agent for solid tumors such as breast and ovarian cancer [1, 2]. However, its clinical utility is often limited by chemotherapy‐induced neuropathic pain (CINP), which manifests as chronic burning, numbness, and glove‐and‐stocking paresthesia, frequently accompanied by motor and autonomic deficits [3]. Approximately one‐third of patients require dose reduction or treatment cessation due to these symptoms [4, 5, 6]. Importantly, many patients with nerve injury‐related pain also develop anxiety and depression. Pain and negative affect often become chronic and mutually reinforcing, thereby leading to a marked reduction in quality of life [7, 8]. Despite extensive clinical characterization, the neural mechanisms that link paclitaxel‐induced neuropathy to persistent affective disturbances remain poorly understood.

Previous studies have mainly focused on peripheral nerve damage caused by paclitaxel, but recent work has begun to reveal central mechanisms of chemotherapy‐induced pain [9]. Persistent chemotherapy pain activates the central amygdala (CeA), promotes corticotropin‐releasing hormone (CRH) release, and strengthens excitatory transmission in hippocampal CA1, forming a “pain‐anxiety” trace [10]. As a major hub integrating pain and affect [11, 12], the CeA receives ascending nociceptive inputs from the spinal cord via parabrachial nucleus (PBN) glutamatergic projections and sends outputs to the hypothalamus and periaqueductal gray to coordinate pain perception and emotional responses [13]. Chemogenetic inhibition of the CeA lowers both chemotherapy‐induced pain and anxiety. In visceral‐pain conditions, PBN‐CeA and basolateral amygdala (BLA)‐CeA transmission are similarly potentiated with upregulated corticotropin‐releasing factor [14, 15]. Whether nociceptive signals generated by paclitaxel‐induced peripheral neuropathy modify CeA function through the PBN‐CeA circuit, thereby driving neuropathic pain and negative affect, remains unclear.

The central amygdala integrates pain and affect through multiple neuromodulatory systems, among which the prodynorphin (PDYN)/κ‐opioid receptor (KOR) pathway is particularly important. PDYN is cleaved into dynorphin peptides (dynorphin A, dynorphin B, α‐neo‐endorphin) that act as high‐affinity endogenous KOR agonists. KOR activation produces robust aversive states, driving dysphoria, avoidance, and anxiety‐like behavior; conversely, PDYN deletion or KOR blockade prevents stress‐induced anxiety [16, 17, 18]. Thus, dynorphin/KOR signaling critically regulates nociception, affect, and stress responsivity.

Genetic evidence supports this role. Individuals carrying the PDYN rs1997794 TT allele, associated with reduced expression, show heightened conditioned fear, impaired extinction, reduced prefrontal activity, and altered amygdalo–prefrontal connectivity [19]. Clinically, elevated dynorphin levels in synovial fluid correlate with treatment‐related pain relief in arthritis patients [20]. Collectively, these findings identify the PDYN/KOR system as a key modulator of pain and negative affect. Whether dynorphin–KOR signaling within the CeA is recruited by ascending PBN input to mediate paclitaxel‐induced pain and affective disturbances remains unknown.

In this study, we used a paclitaxel‐induced rat model to investigate pain–affect comorbidity and the role of the PBN‐CeA pathway. CINP rats exhibited increased CeA activity, and bidirectional optogenetic manipulation of PBN‐CeA projections altered both pain hypersensitivity and anxiety‐like behavior. PDYN expression in the CeA was upregulated in CINP rats, and intra‐CeA KOR blockade with Navacaprant attenuated both phenotypes. Prolonged inhibition of the PBN‐CeA circuit also reduced PDYN transcription. Together, these findings identify the PBN‐CeA pathway and dynorphin–KOR signaling as key contributors to pain and negative affect in CINP, providing a mechanistic basis for targeted therapeutic strategies.

## 2 Materials and Methods

### 2.1 Animals

Male Sprague–Dawley rats ( *Rattus norvegicus* , 160–200 g) were supplied by the Institute of Experimental Animals, Fujian Medical University, Fuzhou, China (production license SCXK(Min)2016–0002). Animals were housed three per cage under controlled temperature (24°C ± 1°C) and 50%–60% relative humidity, on a 12 h light/12 h dark cycle, with ad libitum access to standard rodent chow and filtered water for a 7‐day acclimation period. All procedures were approved by the Animal Care and Use Committee of Fujian Medical University (Approval No. FJMU‐IACUC‐2023‐Y‐1044) and conducted in accordance with the Guidelines for the Care and Use of Laboratory Animals issued by the Ministry of Science and Technology of China (2006) and the ARRIVE 2.0 guidelines. All rats were randomly divided into groups via a random number table. Behavioral, molecular, and histological analyses followed standard protocols. Investigators were blinded to group assignment throughout outcome assessment and data analysis.

### 2.2 Establishment of Chemotherapy‐Induced Neuropathic Pain Model

Chemotherapy‐induced peripheral neuropathy was induced by four intraperitoneal injections of paclitaxel (MCE, HY‐B0015, 2 mg kg^−1^) on days 1, 3, 5, and 7 (cumulative dose 8 mg kg^−1^). For each injection, paclitaxel was diluted to 1 mg mL^−1^ in 0.9% NaCl from a 6 mg mL^−1^ stock prepared in 10% DMSO + 5% Tween‐80 + 40% PEG300. Control rats received an equivalent volume of the same vehicle.

### 2.3 Description of Behavioral Tests

After stable pain hypersensitivity developed, pain‐related behavioral tests were conducted first. The open field test (OFT) and elevated plus maze test (EPMT) were performed sequentially at 24‐h intervals. All animals received behavioral tests in a unified order under identical conditions to avoid order effects and carryover interference. Adequate intervals between tests were also set to alleviate impacts of stress, fatigue, and residual effects. Pain‐related behavioral tests were conducted at 20, 40, and 60 min after drug delivery. Affective behavioral assays were performed 20 min post‐administration on separate days with identical dosing procedures.

### 2.4 Von Frey Test

Mechanical allodynia was assessed as the 50% paw withdrawal threshold (50% PWT) using calibrated von Frey filaments (2–15 g; Stoelting, USA). Rats were acclimated on a wire‐mesh platform for 30 min, and the mid‐plantar surface of the right hind paw was stimulated until the filament bent ≈90° for 2–3 s; withdrawal, shaking, or licking was scored positive. Testing followed the up–down method with a 15 g cutoff. Four reversals after the first positive response were obtained with ≥ 1‐min intervals, and 50% PWT (g) was calculated using 10^(Xf + kδ)^, where δ = 0.224.

### 2.5 Hot‐Plate Test

Thermal sensitivity was assessed on post‐modeling day 8. The thermal pain assay in this study was performed using an XR1100 hot/cold plate analgesia apparatus (Shanghai Xinruan Information Technology Co. Ltd., Shanghai, China). Rats were habituated to the plate at room temperature for 10 min/day for 3 days. Testing was performed on a hot plate (IITC Model 39D) maintained at 52°C ± 0.2°C, with radiant heat applied to the plantar surface of the right hind paw. A 25‐s cutoff was used to prevent tissue damage; nociceptive behaviors included withdrawal, licking, shaking, or sustained lifting. Animals were tested three times with 20‐min intervals, and the median latency was used.

### 2.6 Open‐Field Test (OFT)

Anxiety‐like behavior was assessed in a square arena (100 × 100 × 40 cm). Rats were habituated to the room for 30 min, placed in the center, and recorded for 5 min from above. Locomotion was analyzed using ANY‐maze (Shanghai Mobile Datum, China) to quantify total distance, distance in the central 50 × 50 cm zone, and time in the center. The arena was cleaned with 20% ethanol between trials.

### 2.7 Elevated Plus‐Maze Test (EPMT)

Anxiety‐like behavior was also evaluated in a gray PVC plus‐maze elevated 50 cm, comprising two open (50 × 10 cm) and two closed arms (50 × 10 cm) connected by a 10 × 10 cm center. Rats were handled (1–5 min/day) for 3 days before testing. On test day, animals were habituated for 30 min, placed on the center facing an open arm, and recorded for 5 min with ANY‐maze. Arm entry required all four paws in the arm. The software quantified open‐ and closed‐arm entries and times.

### 2.8 Stereotaxic Surgery and Virus Injection

Rats were anesthetized with 3% isoflurane. After shaving and iodine disinfection, the skull was exposed, cleaned with 30% H_2_O_2_, and leveled at bregma and lambda using a stereotaxic frame (±0.05 mm; RWD, China). According to Paxinos & Watson, coordinates for the PBN (−8.85 AP, ±2.00 ML, −6.50 DV) and CeA (−2.40 AP, ±4.40 ML, −8.00 DV) were targeted. A 0.8‐mm burr hole was drilled, and tracer or optogenetic virus (1.0–1.2 × 10^12^ VG mL^−1^) was injected into the right PBN using a glass micropipette (tip 15–25 μm, mineral oil–backfilled) at 15–20 nL/min (200–300 nL total). The pipette remained for 15 min, was retracted 0.1–0.2 mm for 2 min, and then withdrawn. Corneas were protected with erythromycin ointment, and body temperature was maintained at 37°C with a heating pad.

### 2.9 Optic‐Fiber Implantation

For optogenetic manipulation an optic fiber (200 μm core, 0.37 NA) was implanted to right CeA immediately after virus injection. Three small craniotomies were drilled to form a triangle; one received a 5‐mm stainless‐steel screw, the other two were thinned without dural breach. The fiber was lowered to 0.1–0.2 mm above the injection site and fixed to the skull and screw with zinc phosphate cement, followed by a layer of dental acrylic for reinforcement.

### 2.10 Guide‐Cannula Implantation

For intracerebral microinfusion, bilateral stainless‐steel guide cannulas (26 G; RWD) were implanted above the CeA and secured as described. After 1 week of recovery, rats received a single microinfusion of the KOR antagonist Navacaprant (1 μg/μL) or vehicle (10% DMSO, 40% PEG300, 5% Tween‐80, 45% 0.9% NaCl). Navacaprant dosage was chosen based on published pharmacokinetic data [21]. Under 2% isoflurane anesthesia, a matched injector was inserted into each cannula and 1 μL of solution was delivered at 0.05–0.1 μL/min over 10 min using a microinjector (Shanghai Gaoge Industry and Trade Co. Ltd., China). Injectors were left in place for an additional 10 min to allow diffusion.

### 2.11 Post‐Operative Care and Histology

The incision was sutured and antibiotic ointment applied. Rats received 20,000 IU penicillin i.p. for 3 d and were singly housed. Viral expression was allowed 3 weeks before behavioral testing. At the end of experiments rats were transcardially perfused; coronal sections (40 μm) were examined with a fluorescence microscope to verify injection sites and implant placement.

### 2.12 Optogenetic Experiments

The implanted fiber ferrule was connected to a 473/589 nm DPSS laser through an optical fiber patch cord equipped with a rotary joint, allowing free movement of experimental animals. Blue or yellow light pulses were delivered into the CeA via implanted optical fibers, and subsequent alterations in pain sensitivity and negative affective behaviors were observed. To minimize potential photothermal effects, relatively low light power intensities and intermittent illumination paradigms were adopted. The light output power was calibrated using an optical power meter prior to each behavioral assay, and light intensity was measured directly at the fiber tip. Light was delivered at 20 Hz with a pulse width of 5–10 ms. For pain‐related behavioral tests, a pulsed stimulation protocol was used, with each stimulation lasting 5–10 s and an inter‐stimulation interval of 20–30 s. For long‐duration behavioral tests, including the open field test and elevated plus maze, a cyclic stimulation protocol was used, consisting of 100 s light‐on and 100 s light‐off cycles throughout the 5‐min behavioral session.

### 2.13 Quantitative Real‐Time PCR

Under deep anesthesia, the CeA was microdissected from rat brains. Total RNA was isolated from CeA tissues using TRIzol reagent (Thermo Fisher Scientific, USA). RNA purity and concentration were determined spectrophotometrically by measuring the absorbance at 260 nm and 280 nm. Complementary DNA was synthesized from total RNA using the Evo M‐MLV RT Kit (AG11705, China). qRT‐PCR was performed using a Real‐Time PCR System, with GAPDH serving as the endogenous control for normalization. The primer sequences used in this study are listed in Table 1.

**TABLE 1:** The sequences of primers used in this study.

Gene name	Primer sequence (5′ → 3′)
*GAPDH*	F: ACGGCAAGTTCAACGGCACAG
	R: GAAGACGCCAGTAGACTCCACGAC
*PDYN*	F: AAGGCCAACTCCTTACCAGC
	R: TCCTCGTTGAAATGGAGCGT
*KOR*	F: GAGGCACCAAAGTCAGGGAAGAT
	R: GGATAACAAAGGCAAAGACGAAGA

### 2.14 Immunofluorescence Staining

Rats were deeply anesthetized and transcardially perfused with PBS followed by 4% paraformaldehyde (PFA). Brains were post‐fixed in 4% PFA for 24 h, cryoprotected in 20% then 30% sucrose, and sectioned coronally at 20 μm on a cryostat (Leica CM3050S).

For immunofluorescence, free‐floating sections were rinsed, blocked for 1 h in 0.3% Triton X‐100, and incubated for 48 h at 4°C with rabbit anti‐c‐Fos (1:1000; Cell Signaling), followed by Alexa Fluor 488 secondary (1:500; Abcam) for 2 h at room temperature. Sections were counterstained with DAPI (1 μg/mL), mounted with Fluoromount‐G, and imaged using an FV3000 confocal microscope (Olympus).

### 2.15 Statistical Analysis

The data are presented as mean ± S.E.M and analyzed with GraphPad Prism 8 (GraphPad, USA). The distribution of each dataset was first assessed with the Shapiro–Wilk test. Based on this, between‐group differences were evaluated using an unpaired Student's *t*‐test (for normal distribution) or the Mann–Whitney U test (for non‐normal distribution). Behavioral data across groups and time were analyzed by two‐way repeated measures ANOVA with Bonferroni post hoc test. *p* < 0.05 was deemed statistically significant.

## 3 Result

### 3.1 Paclitaxel Induces Neuropathic Pain and Negative Emotional Behaviors in Rats

**FIGURE 1:**
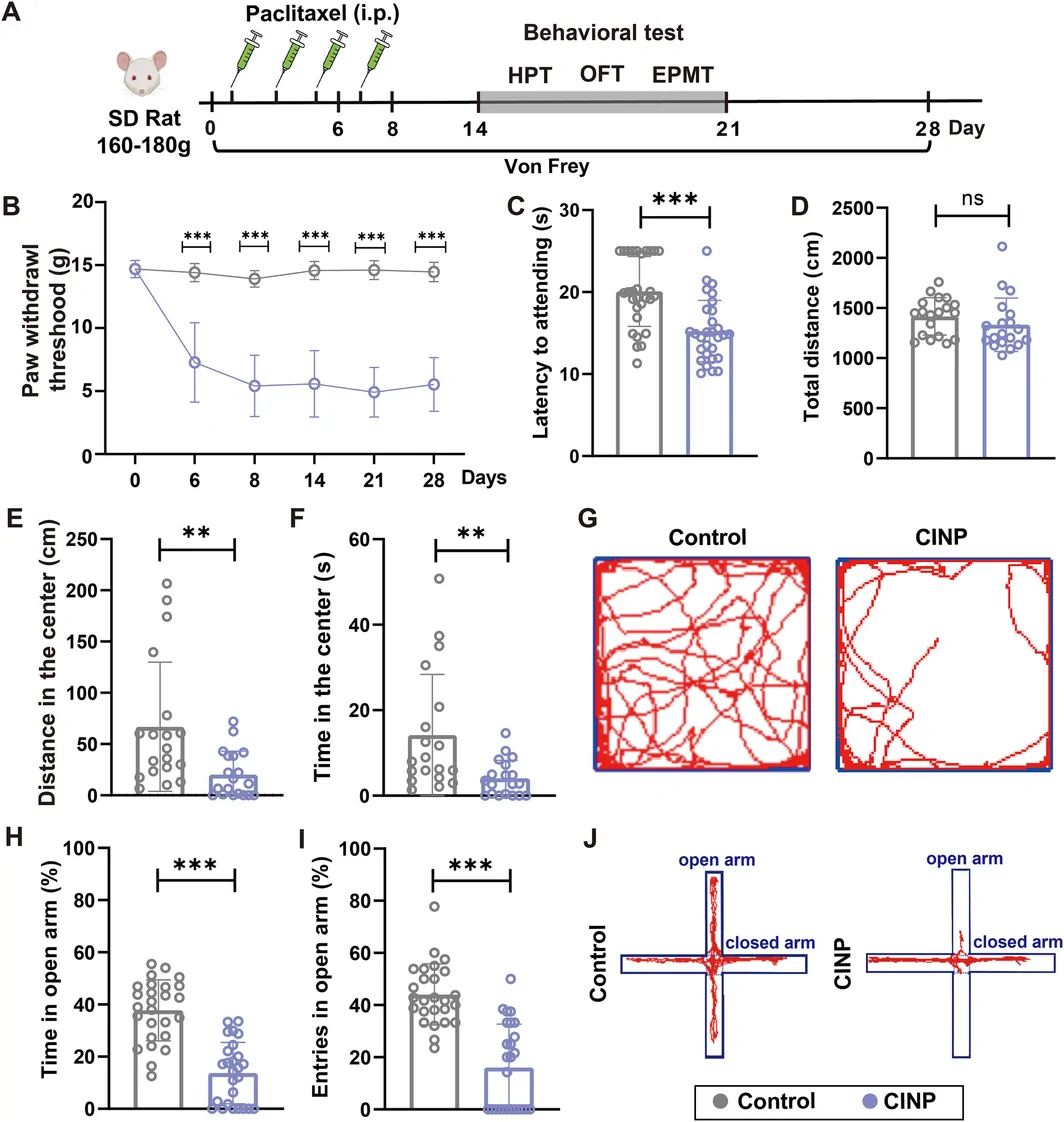
Paclitaxel induces neuropathic pain and negative emotional behaviors in rats. (A) Experimental timeline. (B‐C) Paclitaxel induced mechanical and thermal hypersensitivity in CINP rats. Statistical graph of PWT at different time (B, *n* = 16, two‐way repeated measures ANOVA with Bonferroni post hoc test) and of PWL on day 8 (*n* = 29, unpaired t‐test). (D‐G) CINP rats didn't change the total distance (D) but showed decreased distance in the central area (E) and time in the center area (F) in the open field test (*n* = 19, unpaired t‐test); original trajectory in open field test (G). (H‐J) CINP rats showed decreased time (H) and entries (I) in the open arms of elevated plus maze test (*n* = 26, unpaired t‐test); original trajectory in elevated plus maze test (J). Data are presented as mean ± SEM. **p < 0.01, ***p < 0.001.

To induce the CINP model, rats received intraperitoneal paclitaxel, while control rats received vehicle on the same schedule (Figure 1A). Mechanical sensitivity was assessed using von Frey filaments before injection and on days 6, 8, 14, 21, and 28, while thermal sensitivity was measured using the hot‐plate test. Baseline paw withdrawal threshold (PWT) did not differ between groups (Figure 1B). Following paclitaxel administration, PWT decreased from day 6 and remained significantly reduced on days 8, 14, 21, and 28 relative to controls (*p* < 0.05, Figure 1B). Paw withdrawal latency (PWL) was also significantly reduced in CINP rats (*p* < 0.001, Figure 1C).

Chemotherapeutic agents frequently induce neuropathic pain, which is often accompanied by anxiety‐like behaviors, including pain associated with nerve injury [4, 7]. To determine whether CINP rats displayed similar affective changes, we performed the open‐field test (OFT) and elevated plus‐maze test (EPMT) 1 week after establishing stable pain sensitization. In the OFT, total distance traveled did not differ between groups (Figure 1D), but CINP rats showed significant reductions in both distance traveled and time spent (*p* < 0.001, Figure 1E–G) in the central zone. In the EPMT, CINP rats displayed reduced percentages of time (*p* < 0.001, Figure 1H) and entries (*p* < 0.001, Figure 1I) in the open arms. Together, these findings show that paclitaxel administration produces both sustained mechanical and thermal hypersensitivity and pronounced negative emotional behaviors.

### 3.2 Intra‐CeA Navacaprant Administration Relieves Pain Hypersensitivity and Negative Emotional Behaviors in CINP Rats

**FIGURE 2:**
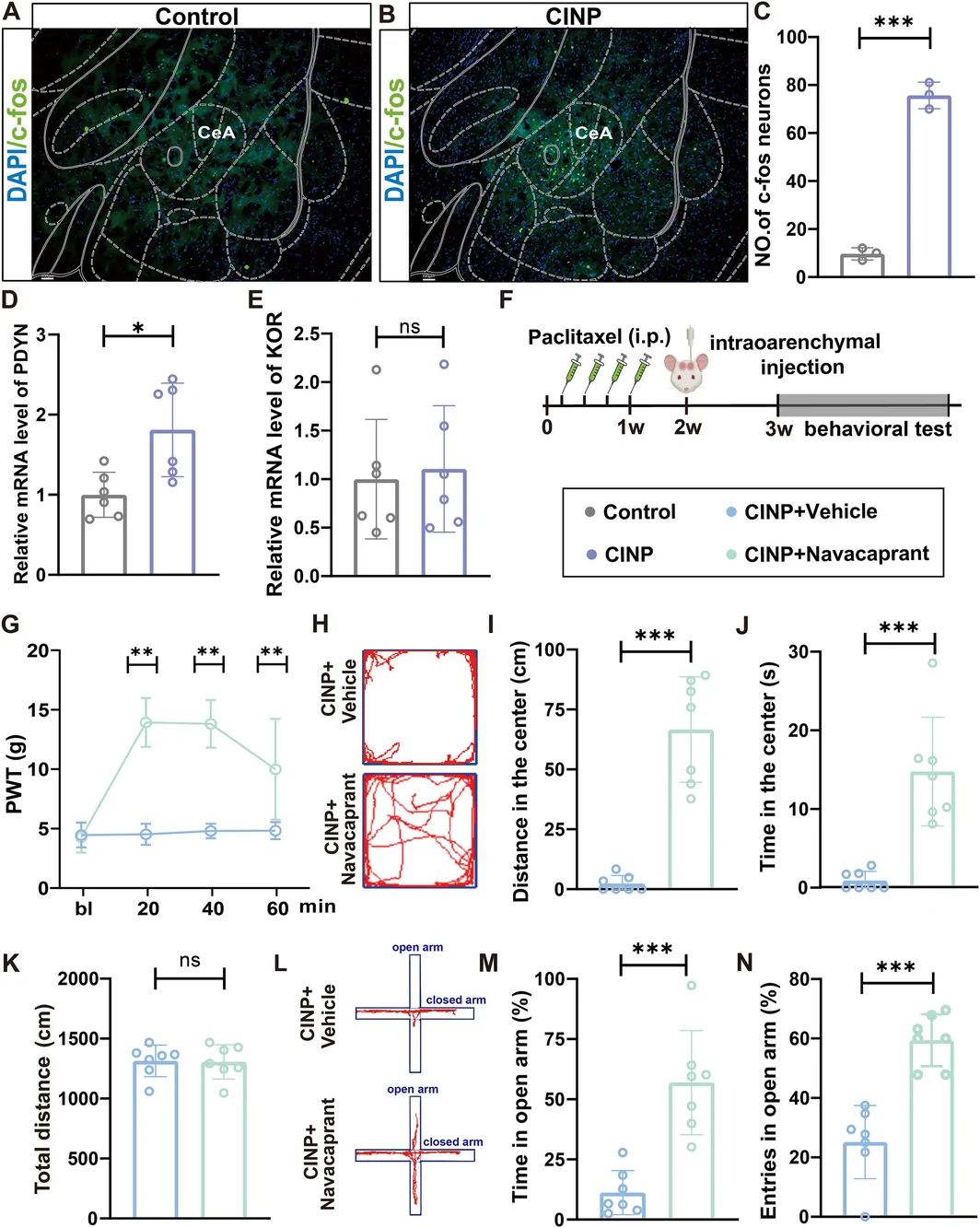
Intra‐CeA navacaprant administration relieves pain hypersensitivity and negative emotional behaviors in CINP rats. (A‐C) Representative immunofluorescence images (A, B) and quantification (C) of c‐fos within CeA in control and CINP rat (*n* = 3, unpaired t‐tes), scale bar, 100 μm. (D, E) The mRNA levels of PDYN and KOR in control and CINP rats (*n* = 6, unpaired t‐test). (F) Schematic timeline of experimental design. (G) Paw withdrawal threshold was significantly increased in CINP + Navacaprant rats compared with CINP + Vehicle controls (*n* = 7 per group). (H‐K) Original trajectory in the open field test (H); Navacaprant‐treated rats exhibited increased central distance (I) and central time (J) withour significant change in total distance (I) in the open field test (*n* = 7 per group). (L‐N) Original trajectory in the elevated plus maze test (L); Navacaprant administration significantly increased time (M) and entries (N) in the open arms of the elevated plus maze test (*n* = 7 per group). Data are presented as mean ± SEM. *p < 0.05, **p < 0.01, ***p < 0.001.

The CeA plays a key role in pain and affective processing. To assess CeA activation in CINP, we examined c‐Fos expression following mechanical stimulation. Rats received three 5‐s von Frey stimuli (10 g; 3‐min intervals), and brains were collected 1 h later. CINP rats exhibited significantly increased c‐Fos–positive neurons in the CeA compared with controls (*p* < 0.001, Figure 2A–C).

The dynorphin–KOR system has been implicated in both nociceptive processing and affective regulation [16, 17, 22, 23], prompting us to examine its status in the CeA. We quantified PDYN and KOR mRNA expression and found increased PDYN levels in CINP rats (*p* < 0.05, Figure 2D), whereas KOR expression remained unchanged (Figure 2E). This pattern suggests increased availability of ligand without a change in receptor abundance, a configuration that could enhance dynorphin‐driven signaling through existing KOR populations.

Given that PDYN acts via KOR, we tested whether pharmacological KOR blockade alleviates CINP symptoms. Navacaprant (1 μg/μL, 1 μL per side) was bilaterally microinjected into the CeA 1 week after modeling, followed by behavioral testing (Figure 2F). Navacaprant significantly increased PWT (*p* < 0.01, Figure 2G). In the OFT, treated rats showed increased distance in the center and time in the center (*p* < 0.001, Figure 2H–J), while total distance was unchanged (Figure 2K). In the EPMT, open‐arm time and entries (*p* < 0.001, Figure 2L–N) were increased relative to controls.

Together, these data show that intra‐CeA KOR blockade with Navacaprant mitigates mechanical hypersensitivity and negative emotional behaviors in CINP rats.

### 3.3 Optogenetic Inhibition of the Right PBN–CeA Circuit Alleviates Neuropathic Pain and Negative Emotional Behaviors in CINP Rats

**FIGURE 3:**
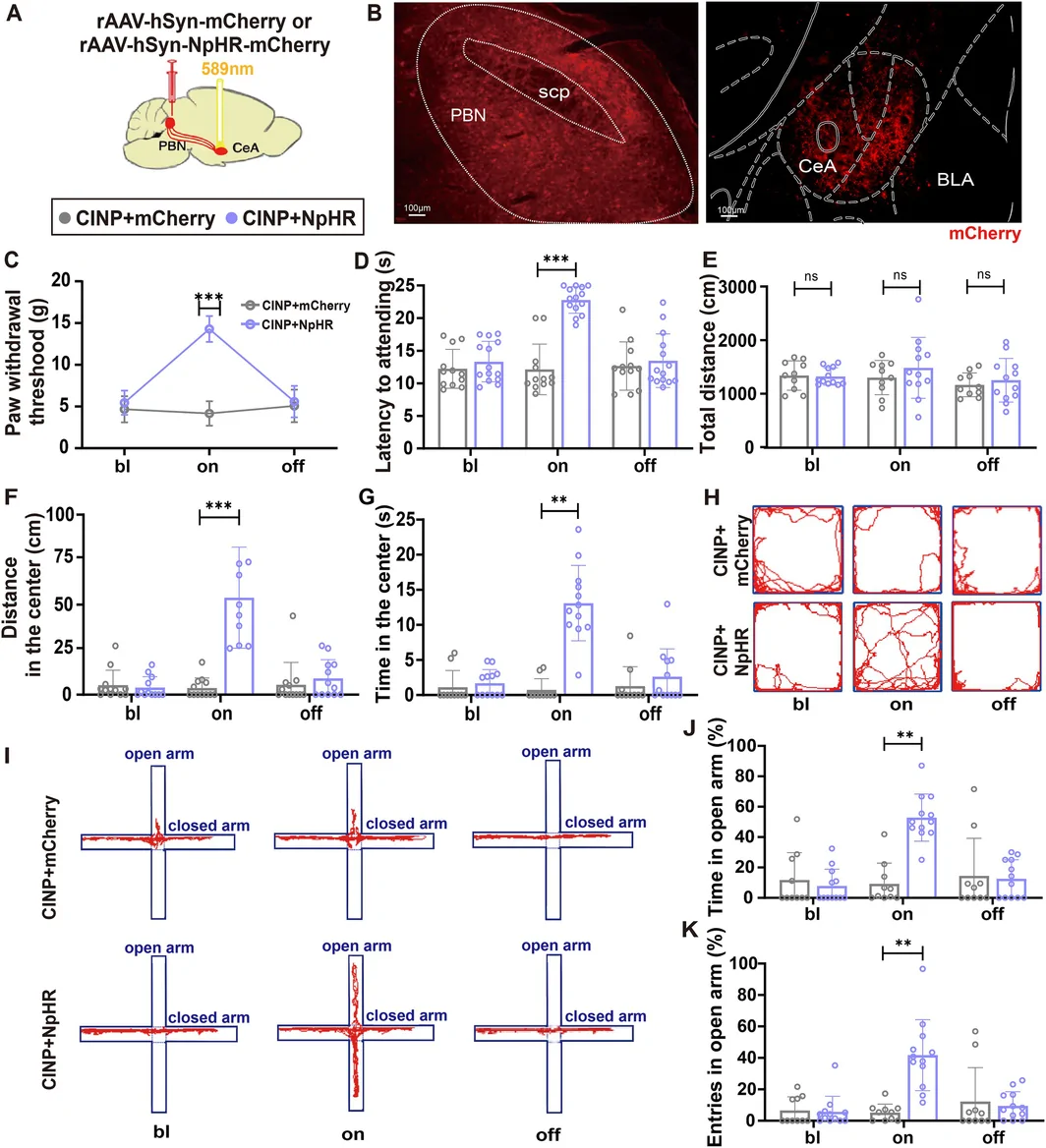
Optogenetic inhibition of the right PBN‐CeA circuit alleviates neuropathic pain and negative emotional behaviors in CINP rats. (A) Schematic diagram of viral injection and optical fiber implantation for PBN‐CeA circuit validation. (B) Representative PBN (right) and CeA (right) mCherry expression images (scale bar, 100 μm). (C‐D) Optogenetic inhibition of the right PBN‐CeA circuit increased PWT (C, *n* = 15–16, two‐way repeated measures ANOVA with Bonferroni post hoc test) and PWL (D, *n* = 12–14, unpaired t‐test) in CINP rats. (E‐H) Optogenetic inhibition of the right PBN‐CeA circuit didn't alter the total distance (E) but increased central distance (F) and central time (G) in the open field test in CINP rats (*n* = 10–12, unpaired t‐test); original traces of the open field test (H). (I‐K) Original trajectory in the elevated plus maze test (I); optogenetic inhibition of the PBN‐CeA circuit increased time (J) and entries (K) in the open arms of the elevated plus maze test in CINP rats (*n* = 10–12, unpaired t‐test). Data are presented as mean ± SEM. **p < 0.01, ***p < 0.001.

Heightened CeA activity and PDYN/KOR signaling in CINP rats suggest a critical contribution of the CeA. Prior work indicates that the right, but not left, CeA is essential for chronic pain [24, 25, 26], and that the right PBN provides dominant nociceptive input during early neuropathic states [27], supporting a functional role for the right PBN–CeA pathway. Viral tracing revealed robust mCherry‐labeled neurons in the right PBN and their terminals in the CeA (Figure 3A,B), confirming a direct monosynaptic projection.

To determine whether this circuit is functionally engaged in CINP, AAV‐hSyn‐NpHR‐mCherry or control AAV‐hSyn‐mCherry was injected into the right PBN, and an optical fiber was placed above the right CeA. Yellow‐light photoinhibition (5 mW, 5 min) significantly increased PWT (*p* < 0.001, Figure 3C) and PWL (*p* < 0.001, Figure 3D) in CINP + NpHR rats relative to CINP + mCherry controls, indicating reduced nociceptive sensitization.

We next tested whether circuit inhibition also attenuates negative emotional behaviors. Following the same photoinhibition protocol, total distance in the open‐field test did not differ between groups (Figure 3E), but CINP + NpHR rats showed significantly greater center distance and center time (*p* < 0.001, Figure 3F–H). In the elevated‐plus maze, CINP + NpHR rats displayed higher percentages of open‐arm time and open‐arm entries (*p* < 0.001, Figure 3I–K).

Together, these data show that silencing the right PBN‐CeA circuit alleviates both nociceptive and negative emotional behaviors in CINP rats, indicating that this pathway is actively engaged in driving pain sensitization and negative affect.

### 3.4 Optogenetic Activation of the Right PBN–CeA Circuit Induces Neuropathic Pain and Negative Emotional Behaviors in Rats

**FIGURE 4:**
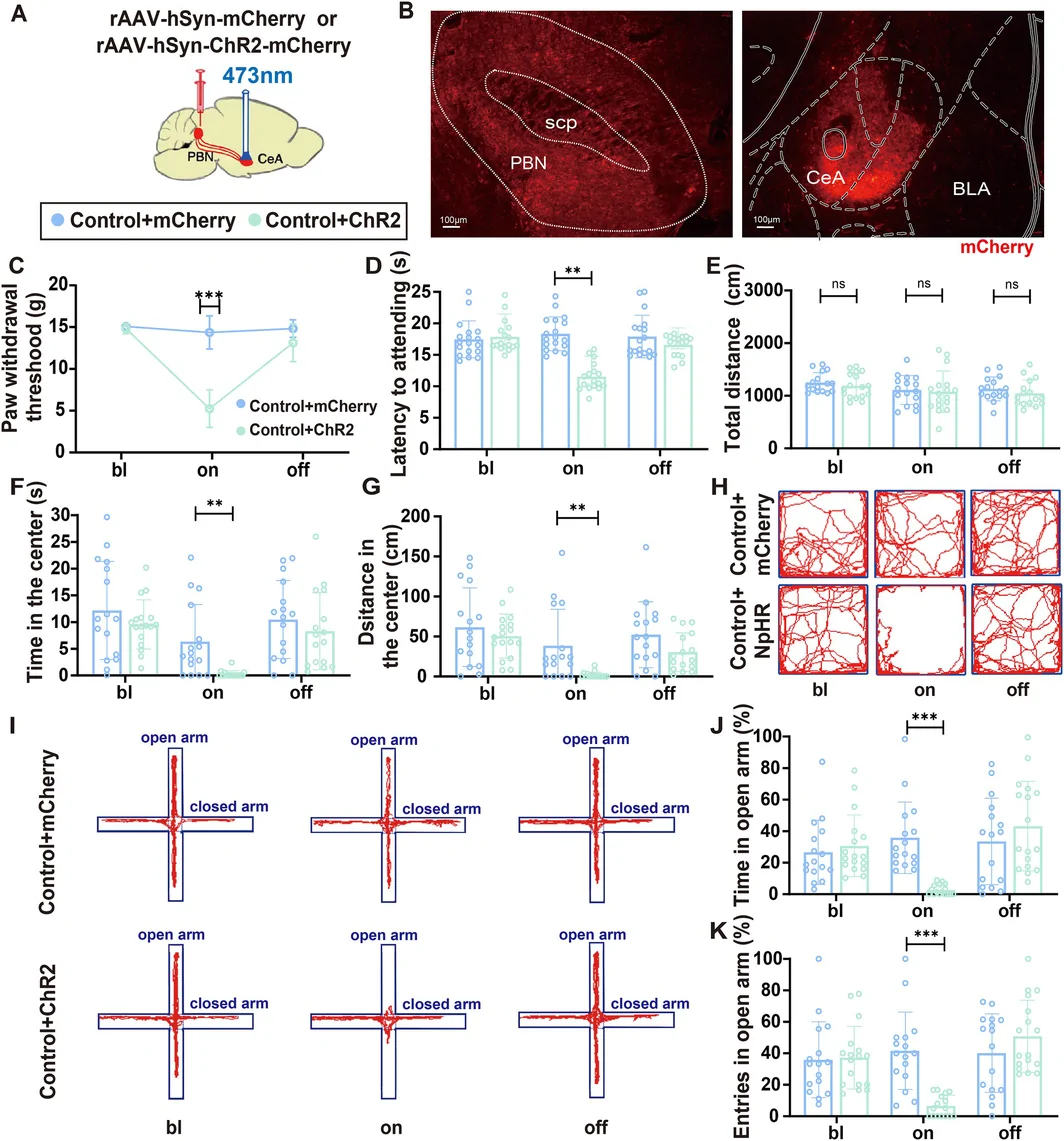
Optogenetic activation of the right PBN‐CeA circuit induces neuropathic pain and negative emotional behaviors in control rats. (A) Schematic diagram of viral injection and optical fiber implantation for PBN‐CeA circuit validation. (B) Representative PBN (right) and CeA (right) AAV expression images (scale bar, 100 μm). (C‐D) Optogenetic activation of the right PBN‐CeA circuit decreased PWT (C, *n* = 15–16, two‐way repeated measures ANOVA with Bonferroni post hoc test) and PWL (D, *n* = 16–17, unpaired t‐test) in control rats. (E‐H) Optogenetic activation of the PBN‐CeA circuit didn't alter the total distance (E) but decreased central distance (F) and central time (G) in the open field test in the control rats (*n* = 16, unpaired t‐test); original traces of the open field test (H). (I‐K) Original trajectory in the elevated plus maze test (I); optogenetic activation of the right PBN‐CeA circuit decreased time (J) and entries (K) in the open arms of the elevated plus maze test in the control rats (*n* = 16, unpaired t‐test). Data are presented as mean ± SEM. **p < 0.01, ***p < 0.001.

Having shown that inhibition of the right PBN‐CeA circuit alleviates pain and anxiety, we next asked whether activation of this pathway in naïve rats is sufficient to induce hypersensitivity. AAV‐hSyn‐ChR2‐mCherry or control virus was injected into the right PBN, and an optical fiber was implanted above the CeA. Blue‐light stimulation (20 Hz, 5 mW, 5 min) activated PBN terminals (Figure 4A), and robust mCherry labeling was observed in the PBN and CeA (Figure 4B). ChR2‐expressing rats showed significantly reduced PWT (*p* < 0.001, Figure 4C) and PWL (*p* < 0.001, Figure 4D), indicating that acute activation of this pathway is sufficient to produce neuropathic pain–like hypersensitivity.

We then examined whether activation of the right PBN–CeA circuit also evokes negative emotional behaviors. Following the same stimulation protocol, total distance in the open field test did not differ between groups (*p* > 0.05, Figure 4E), but ChR2 rats exhibited reduced center‐zone distance and center‐zone time (*p* < 0.001, Figure 4F–H). In the elevated plus maze, ChR2 rats showed lower percentages of open‐arm time and open‐arm entries (*p* < 0.001, Figure 4I–K) than mCherry controls.

Thus, optogenetic activation of the right PBN‐CeA circuit in naïve rats is sufficient to induce both neuropathic pain and negative emotional behaviors, supporting the pathway's critical role in chronic pain and comorbid negative affect.

### 3.5 Prolonged Inhibition of the PBN‐CeA Circuit Down‐Regulates PDYN mRNA Expression in the CeA of CINP Rats

**FIGURE 5:**
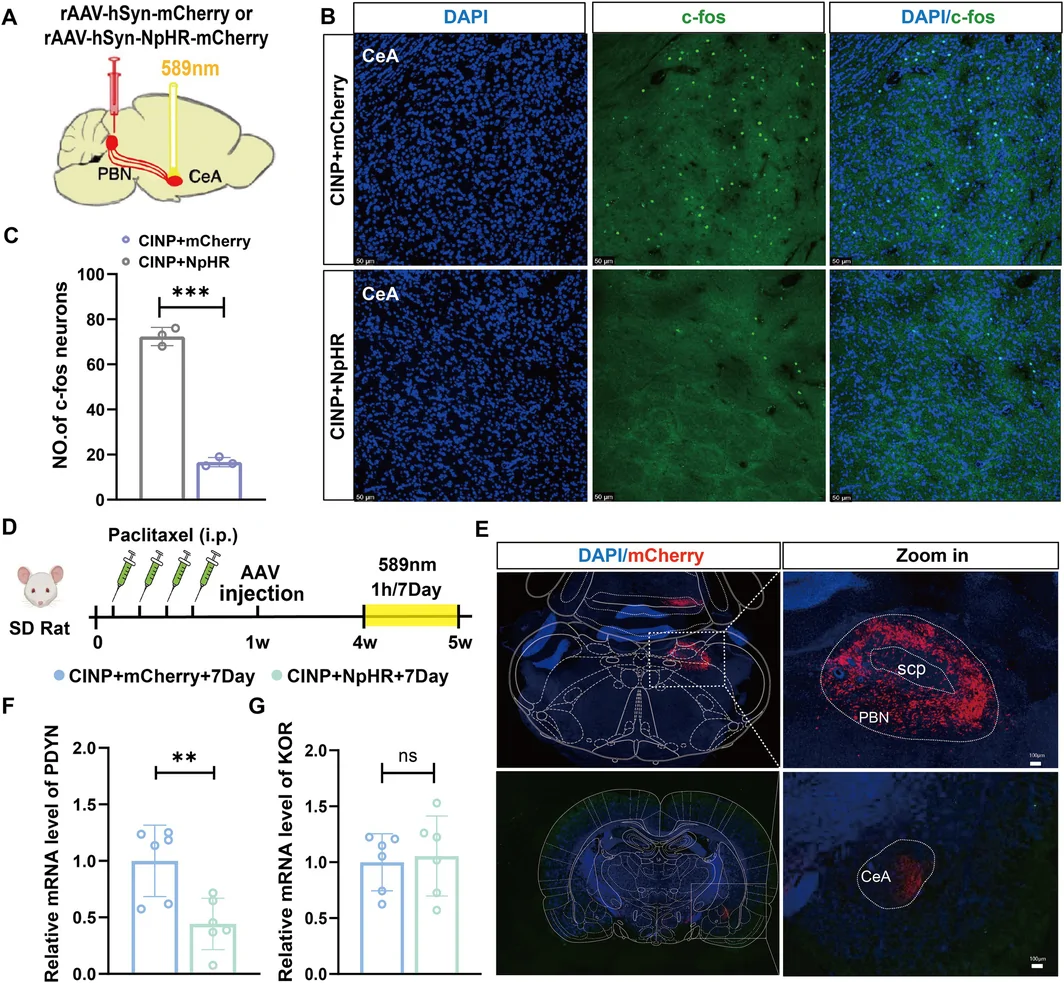
Inhibition of the PBN‐CeA circuit attenuates CeA activity and selectively down‐regulates PDYN expression. (A) Schematic diagram of viral injection and optical fiber implantation for PBN‐CeA circuit validation. (B, C) Representative immunofluorescence images (B) and quantification (C) of c‐fos within CeA in CINP + mCherry and CINP + NpHR rats. Scale bar,100 μm (*n* = 3, unpaired t‐tes). (D) Schematic timeline of experimental design. (E) Representative images of AAV expression in the PBN and mCherry‐labeled nerve terminals projecting to the CeA (scale bar, 100 and 50 μm). (F‐G) The mRNA levels of PDYN and KOR in CINP + mCherry and CINP + NpHR rats. (*n* = 6, unpaired t‐test). Data are presented as mean ± SEM. **p < 0.01, ***p < 0.001.

To determine whether the PBN‐CeA circuit contributes to CeA hyperactivity, we first quantified neuronal activation. Immunofluorescence revealed markedly fewer c‐Fos–positive neurons in the CeA of CINP + NpHR rats than CINP + mCherry controls (Figure 5A,B), with quantification confirming a significant decrease (*p* < 0.001, Figure 5C).

Given this reduction in neuronal activity, we next investigated downstream molecular consequences of prolonged circuit inhibition. As shown in Figure 5D, AAV‐hSyn‐NpHR‐mCherry was injected into the right PBN and an optical fiber was implanted above the CeA. Robust expression in the PBN and mCherry‐labeled terminals in the CeA was observed (Figure 5E). 3 weeks later, CINP rats received daily yellow‐light stimulation (5 mW, 1 h) for 7 days. qPCR revealed a significant decrease in PDYN mRNA in CINP + NpHR rats (*p* < 0.005, Figure 5F), whereas KOR mRNA levels remained unchanged (Figure 5G).

Thus, sustained inhibition of the PBN–CeA circuit reduces PDYN transcription in the CeA, indicating that activity within this pathway regulates dynorphin expression.

## 4 Discussion

In this study, we used a CINP rat model with optogenetic and molecular approaches to evaluate the PBN‐CeA circuit and CeA dynorphin–KOR signaling in pain–anxiety comorbidity. Manipulating the PBN‐CeA projection bidirectionally altered nociceptive hypersensitivity and anxiety‐like behavior, and CINP rats displayed elevated PDYN expression in the CeA, suggesting coordinated circuit and neuropeptidergic contributions to paclitaxel‐induced phenotypes.

Paclitaxel‐induced neuropathy is progressive and often treatment‐limiting, characterized by sensory disturbances that can necessitate dose reduction or discontinuation [28, 29, 30]. A substantial proportion of patients also develop anxiety or depression [31, 32]. Consistent with this, paclitaxel administration in rats produced stable mechanical and thermal hypersensitivity alongside negative emotional behaviors in the elevated plus maze and open field, paralleling patient symptomatology.

Because paclitaxel crosses the blood–brain barrier poorly [33], peripheral nerve injury rather than direct central toxicity likely drives central sensitization and affective disturbances. Emerging evidence indicates that N‐demethylsinomenine (NDSM) exerts analgesic effects in neuropathic pain models primarily via central GABAA receptor α2 subunit (GABRA2) mediation and partially through GABRA3, while exerting concomitant anti‐neuroinflammatory activity [34]. Highlighting the peripheral‐to‐central transition as a potential therapeutic window. Individual susceptibility, sex, and other chemotherapeutic agents may further influence this process. Nonetheless, this model provides a useful platform for investigating neural mechanisms linking chemotherapy‐induced peripheral neuropathy to persistent nociceptive and affective symptoms.

The PBN relays nociceptive input to the CeA via direct glutamatergic projections [35, 36, 37]. Our viral tracing confirmed this connection and showed stronger mCherry fluorescence in the PBN than in the CeA, consistent with evidence that only ~10% of PBN neurons activated by noxious stimuli project to the CeA [38]. In chronic pain models, peripheral injury enhances PBN‐CeA synaptic transmission and neuronal firing [13, 37, 39, 40, 41]. This pathway exhibits lateralized processing, with spinoparabrachial fibers terminating predominantly ipsilaterally [18, 42, 43, 44], the right PBN receives strong spinal input and responds robustly to noxious stimulation, and the right—but not left—CeA mediates chronic pain [24, 25, 26, 27, 45, 46, 47, 48]. These observations motivated our focus on the right PBN‐CeA pathway.

The CeA integrates nociceptive and affective signals, and pain‐related input increases CeA excitability by reducing inhibitory tone and enhancing glutamatergic drive. Inflammatory and neuropathic pain up‐regulate activity markers (c‐Fos, pERK), potentiate synaptic transmission, and recruit PKC/MAPK signaling, promoting hyperexcitability of pain‐facilitating populations [49, 50]. Some studies have shown that microglia within the amygdala regulate stress‐associated mood disorders via selective synaptic phagocytosis [51]. In our study, CINP rats exhibited robust CeA activation. Optogenetic inhibition of PBN terminals increased mechanical and thermal thresholds, reduced anxiety‐like behavior, and suppressed CeA c‐Fos expression, indicating functional dependence on ascending input. Conversely, activation of this projection in naïve rats induced hypersensitivity and anxiety‐like behavior. These findings identify the PBN‐CeA circuit as a key substrate through which paclitaxel‐induced peripheral neuropathy recruits amygdalar processing to generate nociceptive and affective disturbances. The bidirectional manipulation further suggests that reciprocal reinforcement between pain and anxiety amplifies symptom burden, positioning the PBN‐CeA pathway as a central hub in chemotherapy‐induced neuropathic states.

The PDYN/KOR system is widely distributed in the CNS and critically regulates nociception and affective states. Prodynorphin is cleaved into dynorphin peptides (dynorphin A, dynorphin B, α‐neo‐endorphin), which act as high‐affinity endogenous ligands for KOR, in contrast to μ‐ and δ‐opioid receptors that are preferentially activated by endorphins and enkephalins [52, 53]. KOR signaling promotes negative affect and nociceptive sensitization [54]; activation within limbic structures such as the nucleus accumbens induces anhedonia, anxiety, depression‐like behavior, and enhanced pain perception [55], and stress augments KOR activity, whereas KOR antagonists mitigate stress‐induced negative affect [18]. In our study, CINP rats showed increased PDYN—but not KOR—mRNA in the CeA. This pattern may reflect activity‐dependent upregulation of dynorphin, whereas KOR expression remains relatively stable, allowing elevated dynorphin release to drive greater activation of existing receptors without altering receptor transcription. Increased dynorphin‐KOR activation may heighten CeA responsiveness to nociceptive input, sustaining pain sensitization [56]. Consistent with this, intra‐CeA administration of the KOR antagonist Navacaprant alleviated both nociceptive hypersensitivity and anxiety‐like behavior. Moreover, prolonged inhibition of the PBN‐CeA projection reduced PDYN transcription, supporting a functional link between ascending parabrachial input and dynorphin‐KOR signaling in paclitaxel‐induced neuropathy.

Although our data demonstrate a functional role of the dynorphin‐KOR system within the CeA, the precise cellular processes through which this pathway influences nociceptive and affective output are still not fully defined. KOR signaling may modulate distinct CeA neuronal subtypes or engage transcriptional or epigenetic programs during chronic neuropathic states. Moreover, the PBN contains anatomically and functionally distinct subregions. Virus‐infected neurons were observed in both the LPBN and MPBN, suggesting that CINP may engage a broader PBN‐CeA projection rather than a single PBN subregion. The LPBN is commonly associated with nociceptive and affective pain processing, whereas the MPBN is considered important for visceral and interoceptive information processing. Given that chemotherapy‐induced neuropathy is accompanied not only by somatic hypersensitivity but also by systemic discomfort and negative emotional responses, inputs from both the LPBN and MPBN to the CeA may contribute to the observed phenotype. Future studies will be needed to further distinguish the relative contributions of LPBN‐CeA and MPBN‐CeA pathways.

Overall, our study identifies the PBN‐CeA pathway as a critical conduit through which peripheral neuropathy engages central circuits to generate pain hypersensitivity and anxiety‐like behavior, and highlights dynorphin‐KOR signaling as a key modulator of these comorbid symptoms. Targeting this system may offer a promising approach to alleviate the intertwined sensory and affective disturbances associated with chemotherapy‐induced neuropathic pain.

## Author Contributions

Huiqin Chen, Liang Chen, and Yu Chen contributed equally to this work; Huiqin Chen: Writing – Original draft, did the experiments, wrote the paper. Liang Chen: Writing – original draft, did the experiments, wrote the paper. Yu Chen: Data curation, analyzed data. Yin Huang: Did the experiments. Fei Fan: Data curation, analyzed data. YiQian Liu: Did the experiments. Qianli Zhang: Did the experiments. Ying Tang: Scientifically revised the manuscript. Aiqin Chen: Designed the study, scientifically revised the manuscript. Chun Lin: Supervision, designed and supervised the study, writing – review and editing. All authors contributed to and approved the manuscript.

## Funding

This study was supported by the Natural Science Foundation of Fujian Province (2023J01169, 2024J01484, and 2025J01685) and the National Natural Science Foundation of China (82471229).

## Disclosure

Open Access License Statement: This is an open access article under the terms of the Creative Commons Attribution License, which permits use, distribution, and reproduction in any medium, provided the original work is properly cited.

## Ethics Statement

The animal experimental protocol of this study has been reviewed and approved by the Institutional Animal Care and Use Committee (IACUC) of Fujian Medical University, with the approval number FJMUIACUC2023‐Y‐1044. The experiment was conducted in strict accordance with the standards for feeding and use of experimental animals formulated by the Institutional Animal Care and Use Committee of Fujian Medical University, and every effort was made to minimize animal suffering.

## Conflicts of Interest

The authors declare no conflicts of interest.

## Data Availability

The supporting data of this study (including raw experimental data and statistical analysis tables) are available from the corresponding author Chun Lin (E‐mail: chunlin77550@126.com) upon reasonable request.
